# Clustered DNA Double-Strand Breaks: Biological Effects and Relevance to Cancer Radiotherapy

**DOI:** 10.3390/genes11010099

**Published:** 2020-01-15

**Authors:** Jac A. Nickoloff, Neelam Sharma, Lynn Taylor

**Affiliations:** Department of Environmental and Radiological Health Sciences, Colorado State University, Fort Collins, CO 80523, USA; Neelam.Sharma@colostate.edu (N.S.); Lynn.Taylor@colostate.edu (L.T.)

**Keywords:** DNA double-strand breaks, DNA base damage, complex DNA lesions, ionizing radiation, chromatin, genome instability, cytotoxicity, radiation oncology

## Abstract

Cells manage to survive, thrive, and divide with high accuracy despite the constant threat of DNA damage. Cells have evolved with several systems that efficiently repair spontaneous, isolated DNA lesions with a high degree of accuracy. Ionizing radiation and a few radiomimetic chemicals can produce clustered DNA damage comprising complex arrangements of single-strand damage and DNA double-strand breaks (DSBs). There is substantial evidence that clustered DNA damage is more mutagenic and cytotoxic than isolated damage. Radiation-induced clustered DNA damage has proven difficult to study because the spectrum of induced lesions is very complex, and lesions are randomly distributed throughout the genome. Nonetheless, it is fairly well-established that radiation-induced clustered DNA damage, including non-DSB and DSB clustered lesions, are poorly repaired or fail to repair, accounting for the greater mutagenic and cytotoxic effects of clustered lesions compared to isolated lesions. High linear energy transfer (LET) charged particle radiation is more cytotoxic per unit dose than low LET radiation because high LET radiation produces more clustered DNA damage. Studies with I-SceI nuclease demonstrate that nuclease-induced DSB clusters are also cytotoxic, indicating that this cytotoxicity is independent of radiogenic lesions, including single-strand lesions and chemically “dirty” DSB ends. The poor repair of clustered DSBs at least in part reflects inhibition of canonical NHEJ by short DNA fragments. This shifts repair toward HR and perhaps alternative NHEJ, and can result in chromothripsis-mediated genome instability or cell death. These principals are important for cancer treatment by low and high LET radiation.

## 1. Introduction

Cells cope with a tremendous amount of spontaneous DNA damage that arises from naturally occurring reactive oxygen species (ROS), reactive nitrogen and carbonyl species, lipid peroxidation products, the chemical lability of DNA, and other mechanisms [1]. DNA damage is also caused by exogenous agents such as ultraviolet (UV) light, ionizing radiation, and chemicals in air, food, and water, as well as many common cancer chemotherapeutics. Cells experience >100,000 spontaneous DNA lesions each day, and the number of steady state lesions is estimated in the tens of thousands. The majority of DNA lesions are single-strand damage including base damage (i.e., ring opened products), a wide variety of adducts including the common 8-oxo-guanine oxidative damage product, and single-strand breaks (SSBs). Spontaneous SSBs are extremely common, estimated at >10,000 per cell per day [2]. SSBs arise in repair intermediates during base excision repair, incomplete processing by topoisomerase I, and other sources. DNA double-strand breaks (DSBs) are less common. Mammalian cells suffer ~50 DSBs per cell cycle [3,4], largely as a result of replication stress when forks encounter DNA lesions, collide with transcription machinery, or encounter difficult to replicate sequences including fragile sites, sequences that can form G-quadraplexes, and sequences that stably associate with proteins [5,6,7,8]. DSBs are also generated when closely opposed single-strand lesions are processed by base excision repair (BER) or nucleotide excision repair (NER), as these processes create intermediates with SSBs or single-strand gaps [9,10,11,12]. Both isolated and clustered DSBs are induced by ionizing radiation [13].

Decades of reductionist research has revealed hundreds of distinct types of DNA lesions and defined how they are induced and repaired, and their mutagenic potential, genome destabilizing properties, and cytotoxic properties [9,10,11,14]. The many types of “simple” base lesions are repaired by base-excision repair, comprising families of glycosylases, AP endonucleases, and accessory factors, including end-processing enzymes, DNA polymerases, and DNA ligase [10,11]. Bulky adducts are helix-distorting lesions such as UV-induced pyrimidine dimers, and are repaired by nucleotide excision repair (NER), comprising both global NER and transcription-coupled NER [9,15]. DSBs are repaired by non-homologous end-joining (NHEJ) and homologous recombination (HR), each comprising sub-pathways [16,17,18,19].

Although cells contend with vast amounts of spontaneous DNA damage every day, nearly all of these lesions are isolated. Clustered DNA lesions, also referred to as complex lesions or locally multiply damaged sites, have been operationally defined as cases where there are two or more lesions within 10–20 bp (~1–2 helical turns of the DNA). While spontaneous lesions are virtually always isolated, clustered lesions are an important product of ionizing radiation exposure. Specific chemicals such as bleomycin and neocarzinostatin are also able to produce clustered lesions; hence, these types of agents are often described as “radiomimetic”. Thus, in nature, clustered DNA lesions are quite rare, but they can be induced by radiation and a few chemicals. DNA repair pathways are highly efficient at processing isolated lesions to minimize mutagenesis and conserve local genetic sequence information and large-scale structural integrity of the genome. Clustered lesions can display a great deal of complexity as there are many permutations of different types of lesions in clusters, different numbers of lesions per cluster, and different spatial distribution of lesions within clusters. Other potentially relevant parameters that can further add to this complexity are the DNA sequence context, chromatin environment, cell cycle phase, DNA damage signaling systems, and the potential for interference among multiple DNA repair pathways. Given this vast complexity, it is no surprise that clustered lesions pose significant challenges to the repair machinery, and can cause delayed or inaccurate repair, or failed repair and cell death. It is well-established that clustered lesions are more mutagenic than dispersed lesions [12,20,21,22,23,24,25]. Mutations can arise at clustered lesions directly, due to inaccurate repair, and indirectly because persistent clustered lesions are more likely to be encountered by replication forks, causing replication stress-induced DSBs [6,12,22].

**Figure 1:**
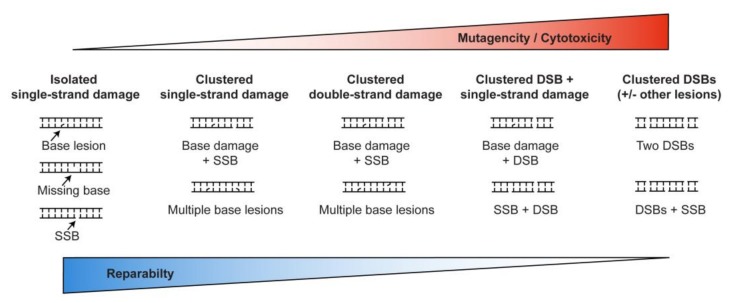
Relationships among DNA damage complexity, reparability, mutagenesis, and cytotoxicity. The triangles above and below indicate lesser to greater biological effects that are inversely proportional to the reparability of isolated vs. clustered DNA lesions.

DSBs are among the most dangerous DNA lesions, in part because unlike single-strand lesions that have an undamaged complementary strand for use as a repair template; DSBs lack such a template. DSB repair can occur without a template, via canonical nonhomologous end-joining (cNHEJ) or alternative NHEJ (aNHEJ, sometimes called microhomology mediated NHEJ). Broken ends at DSBs can also seek a repair template for homologous recombination (HR) repair, such as sister chromatids (limited to S/G2 phases), homologous chromosomes, or homologous sequences (e.g., repetitive elements) elsewhere in the genome. cNHEJ generally results in relatively short deletion or insertion mutations at repair junctions, whereas aNHEJ results in larger deletions as well as chromosome translocations and other large-scale chromosome rearrangements [26,27,28,29,30]. HR repair is usually accurate in the DSB repair region, but associated crossover events pose significant risks of translocations, inversions, deletions and large-scale loss of heterozygosity depending on the configuration of the interacting molecules [29,31,32,33,34,35,36,37]. DSB clusters, induced by ionizing radiation, are especially challenging to repair, and are thus more cytotoxic, mutagenic, and genome destabilizing than isolated DSBs. The picture that emerges is that repair efficiency and accuracy decrease, and mutation and cytotoxicity increase as the complexity of DNA damage increases from isolated single-strand lesions to clustered double-strand lesions (Figure 1).

In this review, we focus on DSB clusters, including their induction by radiation; biochemical and molecular genetic aspects of repair; the cellular consequences of mis-repair or failed repair; and their importance in cancer radiotherapy. We emphasize the biological effects of DSB clusters induced by heavy, charged particle ionizing radiation, such as carbon ions used in advanced cancer radiotherapy, and heavier ions (up to iron) that are ejected from the Sun at relativistic speeds and which pose significant threats to astronauts outside low-Earth orbit. For further information about clustered DNA lesions from the perspectives of radiation physics modeling, chemistry, detection, biology, and genetic consequences, the reader is directed to the following seminal reports and excellent reviews: [13,23,25,38,39,40,41,42,43,44,45,46,47,48,49,50,51,52,53,54,55,56,57,58,59,60,61].

## 2. Radiation-Induced DNA Damage and the Importance of Clustered Lesions

Ionizing radiation damages DNA through direct interactions with DNA and indirectly by producing ROS in the vicinity of DNA, including hydroxyl radicals (•OH) produced by ionization of H_2_O [56]. In mammalian cells, a 1 Gy dose of X-rays induces thousands of single-strand base lesions and SSBs, and ~40 DSBs [48,62]. Although sensitivity to the cytotoxic effects of X-rays varies among mammalian cell types (and during the cell cycle), this level of DNA damage generally kills ~10–20% of cells [48,49,63,64]. There are many types of ionizing radiation, from X-rays and γ-rays which are high energy photons with zero mass, to charged particles ranging from the smallest (protons) to heavy ions such as carbon and iron ions. A useful parameter to describe radiation quality is linear energy transfer (LET). LET is a measure of the density of ionizations along a radiation track, described as the deposition of energy (electron volts; eV) per unit length. X-rays, γ-rays, electrons, and protons are low LET as they deposit energy with ionizations occurring at low density along a track. For example, electron beams used to produce X-rays for external beam radiotherapy have a LET of ~0.2 keV/μm [65]. Because photons lack mass, they have limited interactions with matter in cells and tissues; hence, a significant fraction of X-ray energy passes through the body, producing the familiar X-ray image.

Charged particles, by contrast, have mass and interactions with tissue causes particles to slow and eventually stop at a specific depth (‘stopping point’), determined by the energy of the particle beam. Importantly, the energy deposited along a radiation track by a charged particle increases dramatically near the end of the track, as the particle slows and stops. This concentrated energy deposition at the end of a particle track is termed the “Bragg peak”, initially described by W.H. Bragg in 1903. Protons have a very small mass and a single positive charge, and similar to X-rays are considered low LET radiation; however, it is now understood that the LET of protons increases at the distal end of the Bragg peak [66,67]. Heavy charged particles, like carbon ions and iron ions, are high LET radiation—when these heavier and more highly charged particles slow and stop in the Bragg peak, the density of ionizations is very high. It has long been appreciated that the cytotoxic effects of radiation are, to a significant degree, proportional to LET [68,69,70]. Radiation cytotoxicity is indexed to X-rays, which are defined as having a “relative biological effectiveness” (RBE) equal to 1. As LET increases, so does cytoxicity. For example, the proton RBE is approximately 1.1, as protons are only slightly more cytotoxic than X-rays per unit dose. However, as the proton LET increases at the distal end of the Bragg peak, the RBE in this region may also be higher [66]. Carbon ions are more cytotoxic per unit dose than X-rays by ~2.5- to 3-fold, depending on the cell type; thus, the RBE for carbon ion radiation is 2.5–3. RBE values are important parameters used in radiotherapy treatment planning [66,71,72]. 

The cytotoxic effects of DNA damage are related to both the types of lesions and their spatial distribution (clustering) in DNA. In 1988, J.F. Ward proposed that the cytotoxic effects of (low LET) ionizing radiation reflected its ability to produce clustered DNA damage (originally termed locally multiply damaged sites) [73]. Because a 1 Gy dose of low LET radiation produces thousands of single-strand lesions, comprising base damage and SSBs in a ratio of ~3:1 [48] but only ~40 DSBs, most clustered lesions comprise single-strand lesions on the same strand or on opposing strands; these are called non-DSB clusters. DSBs can be considered a special form of clustered lesion comprising two proximal SSBs on complementary DNA strands. Clustered lesions may also include single-strand lesions near a DSB, the product of at least three ionizations. The energy required to ionize molecules, including DNA, is 20 eV. Thus, three ionizations require 60 eV, which approximates a single energy deposition event in a volume roughly the size of DNA by low LET X-rays or γ-rays [48]. It has been estimated that >80% of complex DNA lesions produced by low LET radiation are non-DSB clustered lesions [56]. This is in good agreement with Ward’s estimate that DSBs comprise ~12% of low LET clustered lesions [48]; it is expected a fraction of DSBs will have associated single-strand lesions forming a complex, DSB clustered lesion. The challenges that cells face when attempting to repair clustered single-strand lesions, as well as their mutagenic and cytotoxic effects, have been well-documented in a number of laboratories [12,20,54,74,75]. It is more difficult to characterize the biological effects of clustered lesions that include a DSB, and only recently have studies been performed to examine the effects of multiple DSBs in clusters (albeit, without associated base damage; see below). Despite these difficulties, it is clear that spontaneous damage is almost always isolated (spatially and temporally) and thus, cells rarely experience clustered lesions (reviewed in [56]). Cells evolved highly efficient DNA repair systems to manage the huge number of (isolated) DNA lesions that arise spontaneously every day. By contrast, because clustered lesions are extremely rare in nature, there has been no selective pressure to evolve systems to repair clustered lesions. Thus, clustered lesions are poorly repaired, accounting for their mutagenic and cytotoxic effects [12,20,54,74,75,76]. Note that this excludes DSBs (a special case of clustered SSBs) for which multiple repair systems have evolved. Spontaneous DSBs are common in S phase, and cells also engage their DSB repair systems to process programmed DSBs for a number of purposes, such as meiotic recombination and immune system development (specifically class-switch recombination and V(D)J recombination) [77,78,79]. Neither spontaneous DSBs nor programmed DSBs occur in clusters. 

## 3. Biological Effects of Clustered DNA Lesions

The critical difference between low LET photons and high LET charged particles is the density of ionizations along a track. In terms of physical-chemical aspects, ionizations (chiefly of H_2_O) induced by different types of radiation are otherwise quite similar. As noted above, clustered lesions are more difficult to repair, hence the increased cytotoxicity of high LET radiation simply reflects the intrinsic weakness of DNA repair systems to process clustered DNA damage. It has been argued that the cytotoxic effects of low LET ionizing are largely due to occasional clustered lesions, rather than the far more numerous isolated lesions [22,49]. Thus, the greater RBE of high LET radiation can be explained by its greater propensity to induce clustered DNA damage, due to its higher ionization density [48,49]. 

Because the ionization products (radiogenic DNA lesions) produced by low and high LET radiation in DNA show similar chemical complexity and differ mainly in lesion density, we can describe these products in the same terms, namely, isolated single-strand damage, non-DSB clustered lesions, and DSB clustered lesions. As ionization density (LET) increases, it is reasonable to assume that at a particular dose, the number of clustered lesions will increase, a point supported by track structure modeling [41,43,44,47,59,61,80,81]. Of particular importance are clustered DSBs. Experiments have directly demonstrated that high LET radiation is indeed more efficient at producing clustered DSBs, yielding small DNA fragments that can be visualized by using atomic force microscopy [82,83]. Experiments have also shown that similar to non-DSB clustered damage, DSB clustered lesions, and specifically clustered DSBs, are repaired less efficiently than isolated damage [22,49,84,85,86]. 

Biochemical experiments provide insight into why clustered DSBs are poorly repaired. cNHEJ is the predominant DSB repair mechanism in mammalian cells, operating throughout the cell cycle [16,18,87,88,89]. cNHEJ initiates with Ku70-Ku80 binding to DNA ends and recruitment of the catalytic subunit of DNA-dependent protein kinase (DNA-PKcs) to produce a DNA-bound, active DNA-PK holoenzyme [19,90]. Although Ku binds efficiently to short (≤32 bp) DNA fragments, the resulting complexes are non-productive as DNA-PKcs is not activated. This inhibition of DNA-PKcs activation was seen with synthetic oligonucleotides, as well as small genomic DNA fragments generated by high LET radiation, indicating that the inhibitory effects are due to fragment length per se, and independent of radiogenic damage at or near broken ends [91]. Activation of DNA-PKcs and subsequent DNA-PKcs autophosphorylation and phosphorylation other targets are essential steps in cNHEJ [92,93], thus the inactive DNA-PK holoenzyme bound to short DNA fragments blocks cNHEJ. Because cNHEJ is the dominant contributor to radioresistance, the evidence that high LET radiation produces short fragments that block cNHEJ provides at least a partial explanation for the greater cytotoxicity (higher RBE) of high vs. low LET radiation. 

**Figure 2:**
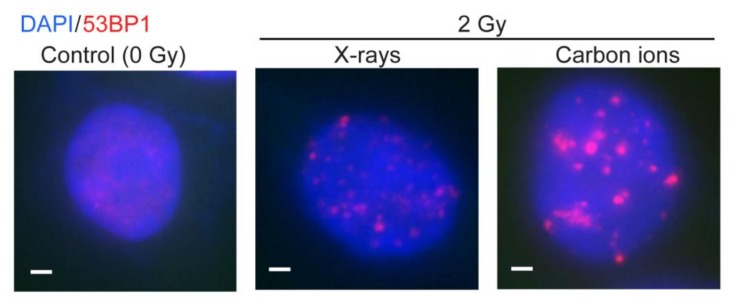
Radiation-induced 53BP1 foci. RKO colon carcinoma cells were mock-treated or irradiated with X-rays or carbon ions. Nuclei were stained with DAPI (blue) and 53BP1 foci (red) were detected by immunofluorescence microscopy. White scale bars are 1 nm. These previously unpublished images were collected during a study comparing the induction of delayed homologous recombination by low or high LET(linear energy transfer) radiation [108].

The inhibitory effect of short DNA fragments on cNHEJ also helps explain several other radiobiological effects of high LET radiation. For example, several studies indicate that DSB repair shifts from the dominant cNHEJ pathway with low LET radiation, to a greater reliance on HR with high LET radiation [94,95,96]. Despite this shift toward HR repair with high LET radiation, at least one study comparing cytotoxicity of low vs. high LET radiation in cells with cNHEJ and/or HR defects indicated that cNHEJ remains the dominant radioresistance pathway [97]. However, this conclusion likely reflects the cytotoxic effects of unrepaired, isolated DSBs, rather than defining the repair pathways that process isolated DSBs vs. clustered DSBs. DNA end resection is enhanced at clustered lesions produced by high LET carbon ions, which is also consistent with the shift toward HR [98]. This shift from cNHEJ to HR when cNHEJ is inhibited is consistent with evidence that these two pathways compete with each other [16,88,99,100,101,102]. DSB repair pathway choice is regulated to a significant degree by end-resection. cNHEJ requires no end-resection, aNHEJ requires minimal end-resection to expose microhomologies near the DSB, and HR requires extensive end-resection to create long, 3’ single-stranded regions bound first by RPA and subsequently by the RAD51 strand exchange protein [17]. 53BP1 and RIF1 are DSB repair proteins that regulate resection and hence DSB repair pathway choice [103,104]. 53BP1 is rapidly recruited to DSBs, where it forms sub-nuclear foci that co-localize with γH2AX, a prominent marker of DSBs [105]. With low LET radiation, 53BP1 foci are usually small and discrete. In contrast, we and others have found that high LET radiation produces 53BP1 foci that are often larger and more persistent, indicative of inefficient repair of clustered DSBs [106,107,108,109,110] (Figure 2). Clustered DSBs induced by high LET radiation have also been implicated in radiation-induced cellular senescence, and persistent changes to chromatin [58,111]; these effects may also be due to the inefficient repair of clustered lesions. 

**Figure 3:**
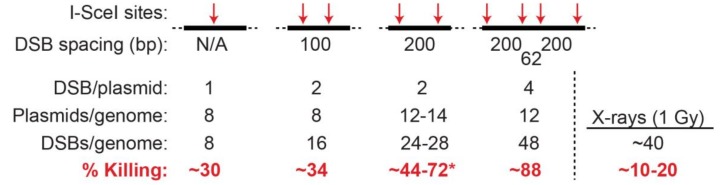
Mimicking the cytotoxicity of high LET radiation with clustered DSBs(DNA double-strand breaks) induced by I-SceI nuclease. Plasmids were created with 1–4 I-SceI nuclease recognition sites spaced from 62–200 bp apart (top; N/A, not applicable). Cell lines were created with 8–14 copies of each plasmid randomly integrated into Chinese hamster ovary cells. Expression of I-SceI induces 8–48 DSBs per genome. Two cell lines were constructed with DSBs located 200 bp apart, with DSB ends either in compatible or incompatible orientation; greater killing was observed with incompatible ends (marked by *). The 4-DSB cluster showed far greater killing per DSB than low LET X-rays. The I-SceI data in this figure were adapted from a report from the Iliakis laboratory [112].

So far, our focus has been on radiation-induced clustered DNA lesions. The Iliakis lab performed an important study to directly address questions about the repair and biological effects of clustered DSBs induced by I-SceI nuclease [112]. Cell lines were constructed with randomly integrated plasmids carrying 1 to 4 I-SceI nuclease recognition sites located from 62- to 200-bp apart, and different cell lines carried 8–14 integrated copies of a particular plasmid. This experimental system was designed to mimic clustered DSBs induced by ionizing radiation. However, since I-SceI induces chemically “clean” (i.e., ligatable) DSBs with 4-base overhangs, DSB induction by I-SceI differs from ionizing radiation as the latter causes chemical modifications at broken ends (so-called “dirty” ends) and it induces large quantities of single-strand damage. I-SceI produces 4-base overhangs, and in this study, the I-SceI sites were arranged such that adjacent I-SceI sites were either compatible (directly ligatable), or in the opposite orientation (incompatible) which require end-processing prior to rejoining (i.e., by cNHEJ). Isolated DSBs and pairs of compatible DSBs separated by 100 or 200 bp caused little cell killing. In contrast, two DSBs separated by 200 bp with incompatible ends caused ~70% cell killing, and ~90% cell killing was observed with a 4-DSB cluster, which are substantially higher than the 10–20% killing caused by ~40 (mostly isolated) DSBs induced by a 1 Gy dose of low LET radiation (Figure 3). 

These results provide direct evidence that clustered DSBs are more cytotoxic than isolated DSBs, and importantly they indicate that these cytotoxic effects are due to DSB clustering per se, and are fully independent of chemically “dirty” radiogenic ends and associated single-strand damage induced by ionizing radiation. There are two other noteworthy results in this study. First, nuclease-induced clustered DSBs show larger and more persistent 53BP1 foci, mimicking the effects of high LET ionizing radiation. Second, cells that survived clustered DSBs displayed chromothripsis (chromosome shattering) that resulted in massive genome instability including chromosome translocations and other large-scale rearrangements [112]. Chromothripsis was originally defined as extensive genomic fragmentation that results in large-scale genome rearrangements that can drive cancer [113,114,115]. However, most chromothripsis events are probably lethal as dicentric chromosomes and other chromosomal rearrangements can cause mitotic catastrophe [116]. In this view, the genome rearrangements associated with chromothripsis appear in rare cells that survive these otherwise catastrophic events. As noted above, small DNA fragments produced by clustered DSBs inhibit cNHEJ, shifting repair toward HR. There is substantial evidence that chromosome translocations are frequently mediated by aNHEJ [27], although other evidence suggests that cNHEJ can mediate translocations in human cells [117]. These pathways may be differentially regulated during the cell cycle [118]. The chromosome translocations induced by clustered I-SceI DSBs may reflect a shift from cNHEJ to aNHEJ due to inhibition of cNHEJ by short DNA fragments. 

## 4. Low and High LET Radiation in Cancer Radiotherapy

The majority of patients treated with external beam radiotherapy receive X-rays, most often intensity-modulated radiation therapy, although high-dose per fraction/hypofractionated stereotactic body radiotherapy is being adopted for certain tumor types [119]. Particle therapy was first proposed in 1946 [120] and the first patients were treated with protons in the 1950s. The first high energy proton therapy center dedicated to clinical practice and capable of treating deep-seated tumors opened in Loma Linda, California, in 1990. There are now >50 proton therapy centers operating in developed countries. The rapid adoption of proton radiotherapy was spurred by the fact that radiation oncologists had considerable experience with low LET X-ray therapy, so the transition to low LET protons was considered safe. Proton therapy also offered benefits to patients with tumors near sensitive tissues (e.g., head and neck, chordoma, and prostate), as well as pediatric patients. Carbon ions (and other moderately heavy ions) were first tested in human clinical trials at Berkeley National Laboratory in the 1970s and 1980s [121], but the project was discontinued when the beam line was repurposed for high energy physics projects. In 1994, the first dedicated clinical carbon ion radiotherapy facility opened in Chiba, Japan. At present, there are 6 carbon ion radiotherapy facilities operating in Japan, two in Germany, one each in Italy and Austria, and three in China. More than 20,000 patients have been treated with carbon ions. The conspicuous absence of carbon ion radiotherapy in the US is explained by several factors. US radiation oncologists were initially concerned about the potentially damaging effects of high LET radiation on normal tissues [122], but these fears have largely been allayed given that serious side effects have generally proven to be as rare (or even rarer) than those seen with traditional X-ray therapy or protons [123,124]. A second factor is cost: advanced X-ray therapy units are ~USD 5 million, and while early proton units were > USD 100 million, they are currently <USD 50 million. The first carbon ion radiotherapy facility in Chiba was ~USD 300 million, but the size and cost of carbon ion units have dropped, with current estimates around ~USD 100 million, plus ~USD 50 million for the building to house the unit. Most carbon ion radiotherapy facilities were constructed with federal government assistance; in Japan, several newer facilities were constructed by public–private partnerships involving, for example, local governments and electric power companies. The third factor that has limited enthusiasm for carbon ion radiotherapy in the US is the lack of data from randomized clinical trials, the gold standard for the adoption of new therapeutic modalities. Such trials are being performed in Germany and elsewhere [125,126,127,128,129], but the results for long-term local tumor control and patient survival will not be available for many years. 

Carbon ion radiotherapy has shown great promise for the treatment of challenging, radioresistant cancers such as head and neck squamous cell carcinoma, chordoma, locally recurrent rectal cancer, and pancreatic cancer [70,123,124,130,131,132,133]. Locally advanced, unresectable pancreatic cancer is a striking example: two-year local control and overall survival are twice the rate with carbon ion radiotherapy as that of the best alternative, intensity-modulated (X-ray) radiotherapy (both in combination with gemcitabine) [130]. It is likely that the improved local control with carbon ions reflects the greater cytotoxicity of high LET radiation. 

## 5. Future Perspectives and Critical Remaining Questions 

**Figure 4:**
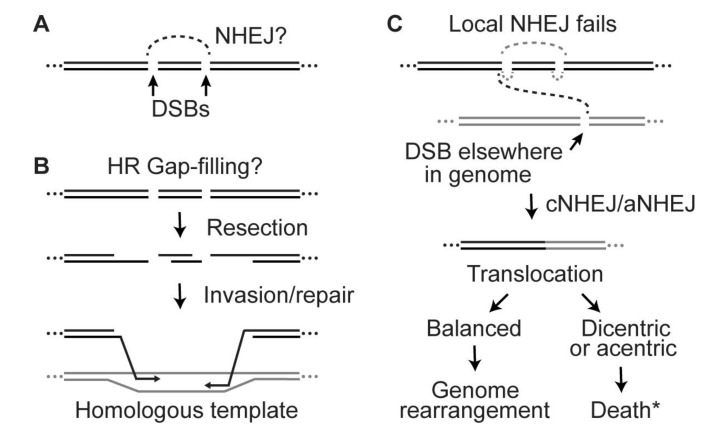
Key questions about clustered DSB repair, illustrated with 2-DSB clusters. (**A**) Short DNA fragments inhibit cNHEJ but it is unclear why distal broken ends, which are termini of long DNA fragments, are not efficiently rejoined (dashed line) by NHEJ. (**B**) Why do the distal ends of a DSB cluster not invade homologous sequences and repair the gap via HR? (**C**) If local NHEJ is delayed or fails at clustered DSBs (grey dashed lines), this may provide time for broken ends to migrate and rejoin with a broken end elsewhere in the genome by cNHEJ or aNHEJ, producing translocations. Balanced translocations are probably survivable events despite the large-scale genome rearrangement. However, unbalanced translocations create dicentric and/or acentric chromosomes that are frequently lethal. “Death*” indicates death plus other possible outcomes, such as stimulation of bridge-breakage-fusion cycles and persistent genome instability [134,135].

The research summarized above provides a useful framework for understanding radiation effects, and it supports both radiotherapy treatment planning and risk assessment for space travel. However, many questions remain unanswered. For example, it is unclear why repair of clustered DSBs does not involve rejoining distal ends. Both cNHEJ and aNHEJ are error-prone, yet these systems are responsible for the majority of DSB repair in mammalian cells. If cells can tolerate NHEJ-mediated insertion and deletion mutations, why does clustered DSB repair not simply delete the (short) DNA fragments by rejoining the distal DSB ends (Figure 4A)? This is especially perplexing given that NHEJ deletions can be as long or longer than the predicted deletions from rejoining distal ends at a DSB cluster. Does the intervening short DNA fragment somehow prevent the distal ends from interacting? It is possible that the NHEJ machinery attempts to preserve the linear order of the DNA sequence by preventing loss of small DNA fragments, despite tolerating sequence errors at re-joined junctions. 

A similar question can be raised with regard to HR repair: why does repair at clustered DSBs not proceed by HR involving strand invasion by distal broken (and resected) ends (Figure 4B)? This could be an efficient and accurate means of gap-filling, effectively restoring the original DNA sequence without involving the intervening short DNA fragment(s). Do small DNA fragments also inhibit HR mediated by distal ends at clustered DSBs? HR requires a homologous template, but often several such templates exist. In the system where clustered DSBs were induced in integrated plasmid DNA by I-SceI nuclease, homologous sequences are present in the other integrated plasmids [112]. However, these may be poorly utilized as HR templates as they are neither sister chromatids (preferred HR templates) nor homologous chromosomes. In the I-SceI system, sister chromatids may not be effective HR templates as they too are subject to cleavage by I-SceI. For HR repair of radiation-induced clustered DSBs, homologous chromosomes or sister chromatids (in S/G2 phase) can serve as repair templates. If clustered DSBs are induced in or near repetitive sequences, repair could be templated from other repeats; note that >50% of the human genome comprises repetitive sequences [136]. Repair from non-sister and non-allelic templates elsewhere in the genome may be restricted, as such interactions pose risks of large-scale chromosomal rearrangements if HR repair is associated with crossovers [31]. Finally, inhibition of cNHEJ by small DNA fragments at clustered DSBs may increase the opportunity for those persistent broken ends to migrate and re-join with broken ends at DSBs elsewhere in the genome, causing translocations (Figure 4C). This idea is related to the observation that DNA end migration and translocations increase when cNHEJ is inactivated by mutation of Ku70 [137]. 

**Figure 5:**
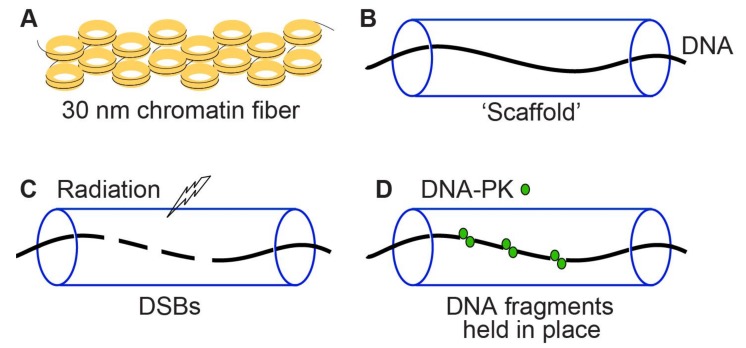
Two potential mechanisms to prevent DSB mis-repair and loss of DNA fragments at clustered DSBs. (**A**) Chromatin consists of DNA wrapped around nucleosomes that are highly compacted into 30 nm fibers and higher order structures. (**B**) Schematic of chromatin shown as a “scaffold” around DNA. (**C**,**D**) Radiation induces clustered DSBs and short DNA fragments that are prevented from mis-rejoining and/or loss by tethering factors such as DNA-PK, and by the chromatin scaffold which may act like a “splint” or “sausage skin” to prevent loss of short DNA fragments.

The MRE11/XRS2/RAD50 and DNA-PK complexes are implicated in tethering DNA ends to promote cNHEJ [138,139]. DNA in chromosomes is highly compacted with histone and non-histone proteins in chromatin. Might a chromatin “scaffold” also act to prevent migration and loss of short DNA fragments at clustered DSBs? This role for chromatin can be described as a “splint” or “sausage skin” model (Figure 5). This model is consistent with observations by Cornforth and Bedford that only ~15% of radiation-induced DSBs can be visualized as breaks in prematurely condensed G1 phase chromosomes [140].

To date, only limited analyses of clustered DSBs have been performed, leaving many unanswered questions about their biological effects. For example, is there an optimal spatial distribution of DSBs to maximize cytotoxicity? Does cytotoxicity continue to increase as the number of DSBs per cluster increases, and if so, does this also depend on the distances between DSBs? A related question is whether the cytotoxicity of clustered DSBs differs if the distance between DSBs is fixed or variable. For example, a 4-DSB cluster might span 150 bp with 50 bp between each DSB, or with 25–50–75 bp spacing. There are obviously many spatial distributions to explore, and while measurements of high LET radiation-induced DSB fragments provide some guidance, radiobiology studies cannot pinpoint specific lethal lesions. Only by using site-specific nucleases can the cytotoxic effects of various DSB spatial distributions be determined. Answers to these questions might drive the development of radiotherapy applications involving mixed low and high LET radiations, such as X-rays, protons, or helium ions plus carbon ions. Although there are technical challenges to produce beam lines for simultaneous (or near-simultaneous) exposures to mixed radiations, the development of such systems is warranted given that radiobiology studies have demonstrated synergistic cell killing with nearly simultaneous exposures to low and high LET radiation [141,142,143], and sequential treatments with different radiations have shown promise in the clinic [127,144,145,146].

There is currently little or no information about clustered damage repair and cytotoxicity in different chromosomal domains, such as euchromatin vs. heterochromatin, or during different phases of the cell cycle. DSB repair in heterochromatin has different genetic requirements than repair in euchromatin [147], and HR is upregulated in S and G2 phases and down-regulated in G1 and M phases [148,149]. Thus, the chromatin environment and cell cycle may differentially affect the repair of isolated and clustered DSBs. Finally, we posit that clustered DNA damage is particularly cytotoxic because cells have not evolved efficient repair systems for these lesions, but is this generally true? Might certain cell types (or cancer cell types) show greater resistance to clustered damage than other cell types?

As noted above, there is clear evidence that clustered DSBs are refractory to repair due to inhibition of cNHEJ and a greater reliance on HR, but it remains unclear what advantage(s) these features might provide, if any. These features may have evolved passively. For example, without selective pressure to repair rare clustered DSBs, cNHEJ machinery may have evolved to efficiently repair dispersed DSBs, perhaps at the expense of inefficient repair of (rare) short DNA fragments. Alternatively, inhibition of cNHEJ by short DNA fragments could have evolved as a means to suppress DSB repair during apoptosis, which causes DNA fragmentation and produces ~140 bp ladders, reflecting the distance between nucleosomes [150]. Thus, inhibition of cNHEJ by short DNA fragments may promote apoptotic death, similar to inhibition of HR during apoptosis by proteolytic destruction of RAD51 [151]. In this view, clustered DSBs might be a signal that the cell has suffered excessive damage and should be eliminated rather than attempting to repair the DSBs with significant mutagenic consequence. Thus, in response to clustered DSBs, cells may be programmed to die by chromothripsis (leading to mitotic catastrophe) and/or by apoptosis. Our understanding of clustered DNA damage induction and repair, and the biological effects of clustered lesions has certainly matured over the past several decades, yet these questions indicate that much remains to be learned about the cellular and genetic consequences of complex DNA lesions.
